# Tropical Medicine

**Published:** 1906-01-20

**Authors:** 


### Tropical Medicine.

Few more important contributions to medical literature have of late years issued from the press than the "Lectures on Tropical Diseases," delivered last August by Sir Patrick Manson at the Cooper Medical College of San Francisco, and now published as an abundantly illustrated volume, replete with information upon the subjects of which it treats. The accounts of the several maladies occasioned by animal and vegetable parasites, the natural histories of these parasites, and the means by which they obtain access to the bodies of their successive hosts are all carefully and lucidly constructed; but, after all, the main interest of the volume is largely in the passages which deal rather with the duties of the legislator than with those of the practitioner. Many years have passed away since the prescience of Sir John Simon pointed out that, as a necessary consequence of increasing facilities for travel, it must be expected that diseases current in the East would become current also in Europe; and Sir Patrick gives detailed accounts of many channels through which, either now or at no remote period of time, great extensions of tropical maladies will be rendered possible if stringent precautions are not speedily taken to prevent them. Of these channels, however, perhaps that from which most danger is to be apprehended is the piercing of the Panama Isthmus; and Sir Patrick is of opinion that, if this work had been accomplished by the French under M. de Lesseps, and before the discovery of the propagation of yellow fever by the Stegomyia mosquito, a frightful extension of that disease would almost of necessity have occurred. Hitherto, he says, there has been little direct or rapid communication between the home of yellow fever—tropical America—and tropical Asia and its islands; but we are now approaching a point in the history of ocean travel and commerce when all this will be changed. A continual procession of ships will, after traversing the yellow-fever area, stream from Panama, and, rapidly passing through the warm Pacific seas, carry freight and passengers to all parts of what is now, as regards the West Indies and Caribbean Sea, the Far East, but which then will be the Near West. The slow sailing ship managed to carry yellow fever to Europe; well then may the fast steamer carry yellow fever to Asia. If stepping-stones are needed, there are Honolulu and the Philippines; and it would be difficult to imagine the extent of the calamity to Asiatic mankind that would follow the introduction of an infected Stegomyia mosquito into such huge distributing centres as Hong Kong or Singapore. What would happen on the introduction of this disease into a thoroughly non-immune, uneducated, insanitary, superstitious, and overcrowded Eastern city—into such a place as Canton or into any of the great centres of population in tropical Asia? There would be a repetition of the depopulation of Uganda by sleeping sickness—a calamity not only terrible in its magnitude, but by which the East would be permanently contaminated, and after which the traveller and trader would have yet another risk and anxiety to face. Sir Patrick believes that a scrupulously administered quarantine on the Panama Canal—a quarantine which need not be irksome to human beings, but which would be absolutely fatal to Stegomyia—would effectually protect Asia against yellow fever. He declares it to be the duty of the United States to carry out this quarantine as one of the responsibilities arising from expanding national influence; and there is every reason to believe, from the activity recently displayed in relation to yellow fever on the Isthmus and from the known source of this activity in the personality of the President, that no duty of this kind is in the least degree likely to be neglected.

